# Type II CRISPR/Cas9 approach in the oncological therapy

**DOI:** 10.1186/s13046-017-0550-0

**Published:** 2017-06-15

**Authors:** A. Biagioni, A. Chillà, E. Andreucci, A. Laurenzana, F. Margheri, S. Peppicelli, M. Del Rosso, G. Fibbi

**Affiliations:** Department of Experimental and Clinical Biomedical Sciences, Section of Experimental Pathology and Oncology, University of Florence, Viale G.B. Morgagni, 50, 50134 Florence, Italy

**Keywords:** CRISPR, Gene therapy, Gene delivery, Genetic engineering, Immune therapy, Oncology

## Abstract

CRISPR (Clustered Regularly Interspaced Short Palindromic Repeats) is a prokaryotic adaptable immune mechanism used by many bacteria and archaea to protect themselves from foreign nucleic acids. This complex system can recognize and cut non-self DNA in order to provide the prokaryotic organisms a strong defense against foreign viral or plasmid attacks and make the cell immune from further assaults. Today, it has been adapted to be used in vitro and in vivo in eukaryotic cells to perform a complete and highly selective gene knockout or a specific gene editing. The ease of use and the low cost are only two features that have made it very popular among the scientific community and the possibility to be used as a clinical treatment in several genetic derived pathologies has rapidly spread its fame worldwide. However, CRISPR is still not fully understood and many efforts need to be done in order to make it a real power tool for the human clinical treatment especially for oncological patients. Indeed, since cancer originates from non-lethal genetic disorders, CRISPR discovery fuels the hope to strike tumors on their roots. More than 4000 papers regarding CRISPR were published in the last ten years and only few of them take in count the possible applications in oncology. The purpose of this review is to clarify many problematics on the CRISPR usage and highlight its potential in oncological therapy.

## Background

### The birth of CRISPR

CRISPR (Clustered Regularly Interspaced Short Palindromic Repeats) system is a prokaryotic adaptable immune mechanism used by many bacteria and archaea to protect themselves from foreign nucleic acids, such as viruses or plasmids [1–4]. The first time CRISPR was ever described was from Osaka University researcher Yoshizumi Ishino in 1987 [5], who accidentally cloned part of a CRISPR together with the *iap* gene, his target of interest and its function was not cleared at the time. Later on, in 1993 researchers of Mycobacterium tuberculosis in the Netherlands published two articles about a repeat cluster in this bacterium that was named “direct repeat (DR)” region. These researchers recognized the diversity in the composition of repeat cluster spacers [6]. At the same time, the later called CRISPR was also observed in the archaeal organism Haloferax mediteranii and its function was studied by Francis Mojica at the University of Alicante, Spain [7]. Anyway, the real begin of CRISPR history, is in 1997 when Ruud Jansen at the University of Utrecht, recognized a similarity among the structure of the *iap* repeats of E. coli, the DR region of M. tuberculosis and the repeat cluster of H. mediteranii, defining these features as members of the CRISPR family. From that discovery numerous CRISPR’s were recognized in the whole genomes of bacteria and archaea that were published, indicating that CRISPR is a universal feature of prokaryotes. A major addition to the understanding of CRISPR came with the observation that the repeat cluster was accompanied in the prokaryotic genomes by a set of highly conserved homologous genes, the CRISPR associated or Cas genes. Four Cas genes (Cas 1 to 4) were recognized and the Cas proteins showed helicase and nuclease motifs, suggesting a dynamic role of these proteins in the CRISPR machinery. For many years CRISPR remain a “mystery item” until 2005, when three independent research groups showed that some CRISPR spacers are derived from phage DNA and extrachromosomal DNA such as plasmids [8–10]. Indeed, the spacers are only fragments of DNA gathered from viruses that previously tried to attack the cell. The source of the spacers was a sign that the CRISPR/Cas system could have a role in an adaptive immunity in bacteria. In 2008, Brouns et al. identified a complex of Cas protein that in E. coli cut the CRISPR RNA within the repeats into spacer-containing RNA molecules [11], which remained bound to the protein complex. In the same year, Marraffini [12] showed that a CRISPR sequence of Staphylococcus epidermidis targeted DNA and not RNA to prevent conjugation. A 2010 study provided direct evidence that CRISPR-Cas cuts both strands of phage and plasmid DNA in S. thermophiles [13]. In 2012 Jinek [14] showed that the core CRISPR/Cas9 mechanism is based on a dual-RNA structure that directs the Cas9 endonuclease to introduce site-specific double-stranded breaks in target DNA. This discovery broke up the old technology such as TALEN, Meganucleases and ZFNs, demonstrating that the guide RNA could be easily engineered as a single transcript to target and cleave any dsDNA sequence of interest. We had to wait until 2014 to see the first example of use as a tool for editing the genome, when Hsu et al. [15] manipulated the resistance of S. thermophilus to phage by adding and deleting spacers whose sequence matched those found in the phages tested. Finally, in 2015 there was the first attempt in editing human embryos [16] showing that even if promising, we are still far from any clinical use of CRISPR technology in embryos, triggering also a controversial ethical debate.

## The mechanism of action

**Fig. 1:**
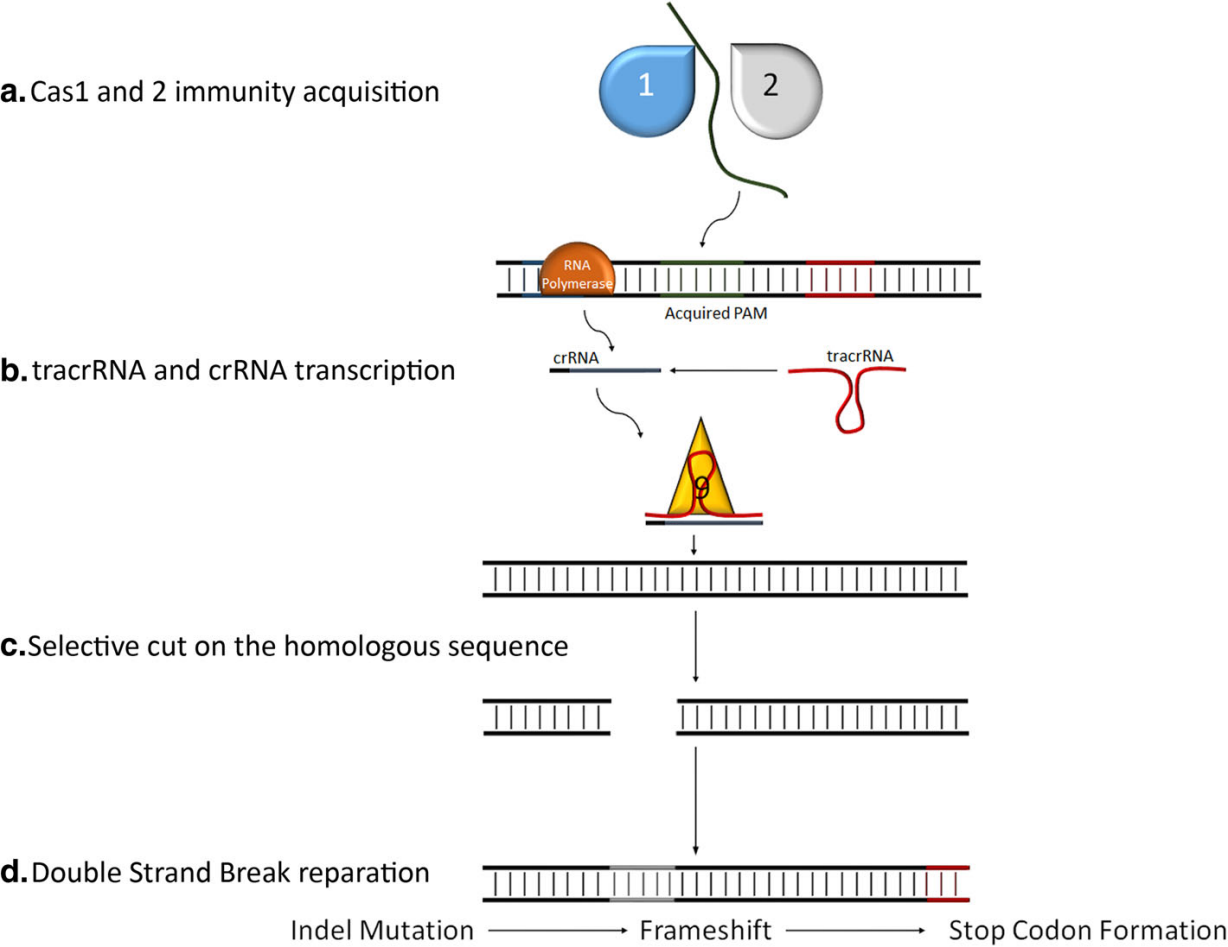
Type II CRISPR mechanism of action. Foreign DNA is cut and acquired by Cas1 and 2 between CRISPR repeat sequences (**a**) forming PAMs. Then a RNA Polymerase transcribes part of the CRISPR repeat and part of the PAM generating a crRNA (**b**) that hybridize with a tracrRNA and reach a homologous target sequence on the genomic DNA (**c**). Cas9 performs a DSB that it is repaired with a NHEJ causing indel mutations and so probably a premature stop codon (**d**)

The CRISPR system can be found on both chromosomal and plasmid DNA. Type II CRISPR incorporate sequences from invading DNA between CRISPR repeat sequences thanks to Cas1 and Cas2 [17]. These regions that are complementary to the foreign DNA are called Protospacers. Transcript from part of these regions are processed into CRISPR RNAs (crRNAs) and hybridizes with a second RNA called transactivating CRISPR RNA (tracrRNA) [18]. This complex of crRNA and tracrRNA binds a nuclease and helicase protein called Cas9. Protospacer-encoded portion of the crRNA directs Cas9 to cleave complementary target-DNA sequences, if they are adjacent to short sequences known as protospacer adjacent motifs (PAMs). PAMs are very important to the recognition of self and non-self DNA because are presents only in the foreign DNA sparing the CRISPR mechanism to delete itself. Indeed, protospacer sequences incorporated into the CRISPR locus are not cleaved presumably because they are not next to a PAM sequence. This prokaryotic system has been adapted to be used in vitro, merging the crRNA with a part of the tracrRNA in a hybrid called guide RNA (gRNA). Twenty nucleotides at the 5’ end of the gRNA (corresponding to the protospacer portion of the crRNA) direct Cas9 to a specific target DNA site using standard RNA-DNA complementarity Watson-Crick base-pairing rules. The site to be cleaved must lie immediately 5’ of a PAM sequence, although recognition at sites with alternate PAM sequences could be possible, although at less efficient rates [19–21] (Fig. 1). Cas9-induced double strand breaks (DSBs) are commonly repaired exploiting the NHEJ (Non Homologous End Join) mediating indel mutations as well as inducing HDR (Homologous Directed Repair) by providing single-stranded oligonucleotide acting as a donor template. In the error-prone NHEJ pathway, the ends of DSB are processed by endogenous DNA repair machinery and rejoined, which results in random indel mutations at the site of junction which can result in the frameshifts within the coding region of a gene and can cause the creation of a premature stop codon, knocking out the target gene. Alternatively, when a repair template in the form of a single-stranded oligodeoxynucleotide (ssODN)is supplied, the HDR pathway allows high fidelity and precise editing [22].

## Cas9 variants

**Fig. 2:**
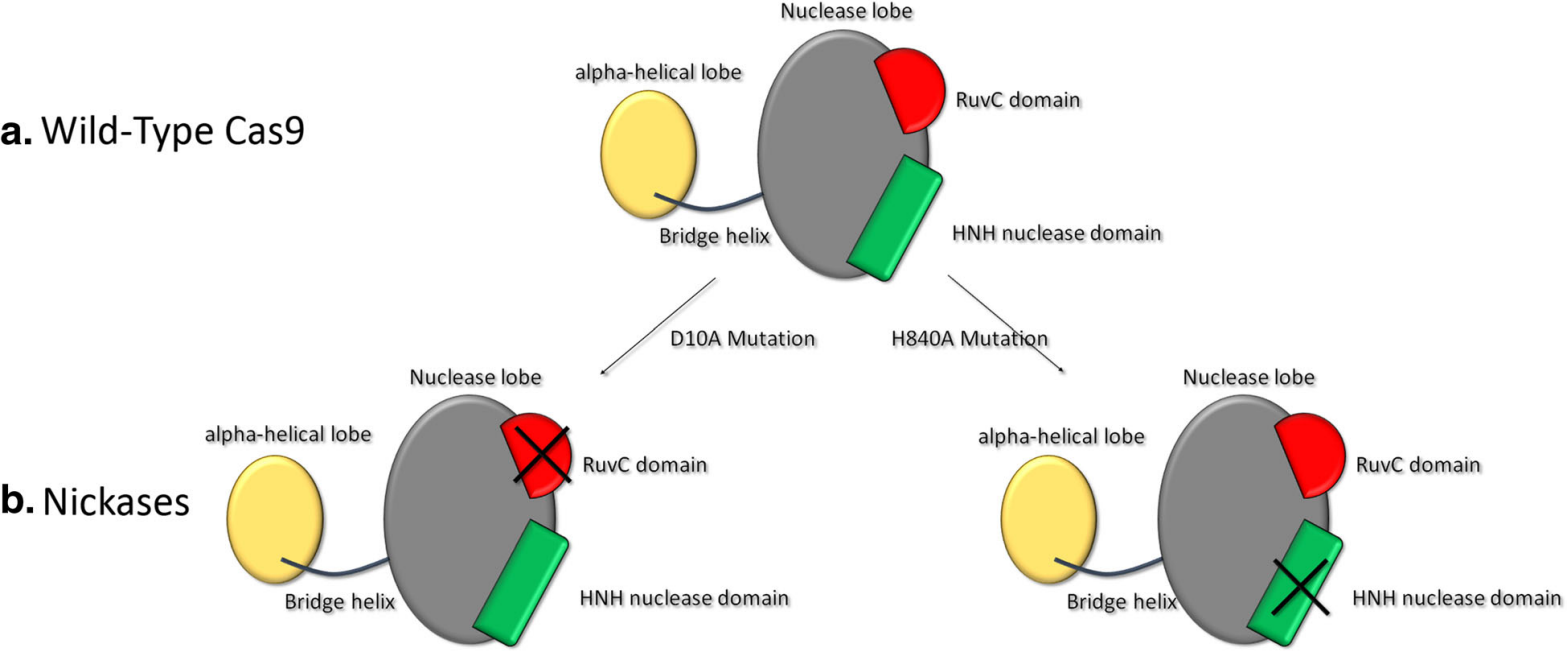
Cas9 structure. The alpha-helical lobe and the nuclease lobe composed by, RuvC and HNH domain. The D10A and the H840A mutations in these last two domains cause the loss of ability to perform a DSB making only a single nick per strand. These particular Cas9 are commonly called Nickases

Cas9 is a bi-lobed architecture protein with the gRNA nestled between the alpha-helical lobe and the nuclease lobe. These two lobes are connected through a single bridge helix. There are two main domains located in the multi-domain nuclease lobe: the RuvC, which shares an RNase H fold structure with other nucleases in the retroviral integrase superfamily [23], which cleaves the non-target DNA strand, and the HNH nuclease domain, that has a ββα-metal fold that comprises the active site, which cleaves the target strand of DNA [24]. The gRNA base paired with target ssDNA is anchored by Cas9 as a T-shaped architecture. The nuclease also consists of a recognition lobe (REC) that matches the target sequence in the host DNA. Several Cas9 mutants including REC domain deletion and residues mutations in the bridge helix (BH) domain have been tested to improve its efficiency and to find other useful “side effects”. REC and BH mutants show lower or none activity compared with wild type, which indicate these two domains are crucial for the gRNA recognition and stabilization of the whole complex. Normally Cas9 performs a double strand break in the target DNA site, while introducing a D10A or H840A mutation into the RuvC- or HNH-like nuclease domains results in the generation of a single cut [25] (Fig. 2). These mutants also known as Nickase have also been shown to be useful for genome editing. Nickase cut either the complementary or non-complementary DNA target strands, respectively, in vitro.

## Off target effect risks and possible solutions

Even if very efficient, this system is not completely immune to errors, so understanding the possible weak sides could be helpful to prevent all potential off-target effects. Recently, a number of studies have examined potential off-target sites that differ from one to six positions from the on-target site in human cells [26, 27]. To prevent these effects, it has been suggested that higher GC content at the RNA:DNA interface might potentially help to stabilize binding the hybridization, indeed high rates of mutagenesis have been observed for off-target sites with as little as 30% matched GC content [24]. Moreover, we also do not know how genomic and/or epigenomic context might affect the frequency of cleavage. Although some initial evidence suggests that DNA methylation does not inhibit Cas9-based genome editing [28], it seems plausible and likely that chromatin structure could play a role in off-target site accessibility. One potential strategy to reduce off-target and improve specificity involves the use of paired nickases in which adjacent off-set nicks are generated at the target site using two gRNAs and Cas9 mutant. Paired Cas9 nickases, targeted to sites on opposite DNA strands separated by 4 to 100 bp, can efficiently introduce both indel mutations and, in case a single-stranded DNA oligonucleotide donor template is provided, can induce HDR events in mammalian cells [29, 30]. However, the second gRNA can itself induce its own range of off-target mutations in the genome as multiple studies have shown that single monomeric Cas9 nickases can function on their own to induce indel mutations at certain genomic loci, perhaps because an individual nick might be converted to a DSB when a replication fork passes through the locus [31, 32]. The existence of these off-target effects and our inability to identify these alterations on a genome-wide scale mean that researchers need to account for the potentially confounding effects of these undesired mutations. The best and easiest strategy to rule out off-target mutations is the use of complementation, reintroducing a wild-type gene, which can be used to confirm the effects of knockout. Another possibility is to use gRNAs targeted to different sites. Presumably, each gRNA will be expected to have a different range of off-target effects and therefore if the same phenotype is observed with each of these different gRNAs it would seem unlikely that undesired mutations are the cause.

## CRISPR delivery possibilities

There are several methods to deliver all the components of CRISPR machinery. In cultured mammalian cells, can be used electroporation, nucleofection and Lipofectamine-mediated transfection of non-replicating plasmid DNA to transiently express Cas9 and gRNAs. Lentiviral vectors have also been used to constitutively express Cas9 and/or gRNAs in cultured human and mouse cells with higher efficiency [33–35]. Another useful delivery system is combining gRNA and an exogenous Cas9 conjugated with cell penetrating peptides (CPP) or encapsulated in nanoparticles [36, 37]. For most applications, transient expression of gRNAs and Cas9 is typically sufficient to induce efficient genome editing. Indeed, after expression and selection plasmid expressing CRISPR machinery is usually lost, avoiding that extended persistence in the cell will lead to increased frequencies of off-target mutations.

## Clinical and therapeutically uses

The advent of CRISPR has not only marked a new era for the in vitro genome editing, giving us a powerful tool to better understand the fine mechanisms at the base of molecular biology but has also revolutionized the “personalized” or “precise” medicine therapy. Many diseases in fact, hide a genetic origin and following the central dogma of molecular biology, we can precisely edit those mutations that cause the disease to restore the expression of the “healthy” form of the protein. As recently reported by three separate research groups [38–40], gene therapy using CRISPR technology for Duchenne muscular dystrophy is ongoing clinical trials showing very positive results both in adult muscle differentiated cells and muscle stem cells. CRISPR approach for treatment of Cystic Fibrosis as well, despite being in its early stages, is very promising. Indeed, stem cells can be corrected by delivering the therapeutic agents into the airways or, alternatively, generating CFTR-corrected stem respiratory epithelial cells and subsequently be administered to the patients [41]. Methods for efficient delivery and expression of CRISPR-Cas system components will undoubtedly need to be optimized for each particular cell-type or organism to be modified. Collectively, these advances will be important for research use and therapeutic applications. Strategies for shifting the balance away from NHEJ-mediated indel mutations and toward HDR-driven alterations remain a priority. Although high rates of HDR can be achieved with the CRISPR and single-stranded DNA oligonucleotides, competing mutagenic NHEJ also occurs simultaneously. One of the drawbacks to developing an approach to improve the HDR:NHEJ ratio is that inhibition of NHEJ is likely to be poorly tolerated by most cells, given its central role in normal DNA repair. For therapeutic applications seeking to exploit HDR, reduction or elimination of competing NHEJ will be crucially important. Another promising therapy is to eliminate viral infection from the host genome using CRISPR. Human Papillomaviruses (HPVs) are today the main responsible for cervical carcinoma and anogenital cancers. Two research groups in 2014 used for the first time CRISPR to induce indel mutations in the viral genes encoding for E6 and E7 proteins in human cells [42, 43]. These proteins, inactivating respectively p53 and pRb, drive infected cells to an abnormal proliferation leading to tumor transformation. CRISPR capability to perform a selective knock out of viral genes is being use in Hepatitis B virus (HBV) as well as in HPVs. Indeed, several researchers designed sgRNAs targeting the HBV core and HBsAg proteins to reduce HBV-related symptoms and to treat HBV-associated disease. The most recent one, is the approach used by Zhen et al. [44] who targeted the HBsAg and HBx-encoding region of HBV, both in vitro and in vivo. HBsAg levels in the cultures media of cells and in the sera of mice were reduced as well as the HBV DNA levels and HBsAg protein expression in mouse livers. Epstein-Barr virus (EBV) is another etiologic agent capable to drive cancer-leading mutations, causing the Burkitt’s lymphoma and the nasopharyngeal carcinoma. While Wang and Quake designed several sgRNAs targeting the nuclear antigens EBNA1, EBNA3C and the latent membrane protein 1 (LMP-1) [45], Yuen et al. [46] used two sgRNAs targeting the promoter region of BART miRNA, to reduce the proliferation and to promote the decline in viral load as well as restoration of the apoptosis pathway in infected cells. As demonstrated by Wang et al. [47], Cas9 can knockout viral sequences of HIV-1 in mammalian cells causing the activation of NHEJ repairing system and generating some indel mutations that are potentially lethal for the virus. So while in some cells the virus is easily eradicated, in others some indel mutations are refractory to recognition by the same gRNA as a result of changing the target DNA sequences, leading to the emergence of replication competent viruses that are resistant to Cas9/gRNA. More recently, Chaoran et al. used an all-in-one adeno-associated virus (AAV) vector to deliver multiplex sgRNAs targeting four different viral structural genes and the Staphylococcus aureus-derived Cas9, demonstrating that this strategy greatly reduces the potential of HIV-1 escape and increases the possibility of HIV-1 excision despite the continuous mutations in the clinical HIV-1 patients’ population [48]. Many other therapies are currently ongoing to cure disease such as haemoglobinopathy, β-thalassaemia, Leber congenital amaurosis, haemophilia giving hope to all patients that could not count on alternative therapy ways [49–51]. Even on cancer research there are some encouraging studies. In the last few years, research focused especially on those types of cancer that are untreatable with standard chemo- or radiotherapies and in particular the lung cancer, which is one of the most fatal and, even if a lot of efforts have been spent, is still the main cause of cancer-related deaths. Immunotherapy has emerged as a promising way to treat lung cancer using therapeutic vaccines and targeting the specifically cytotoxic T lymphocyte associated protein 4 and programmed death receptor 1 (PD-1) pathways [52]. Lu You, an oncologist at Sichuan University’s West China Hospital in Chengdu, started testing modified immune T cells treating non-small cell lung cancer for patient whose chemotherapy, radiation therapy and other treatments have failed [53]. These T cells, which are PD-1 KO, once reintroduced in patients will home in the tumor activating the immune response and hopefully eradicating tumor cells. Indeed, PD-1, also known as CD279, and its pathway is involved in T-cells regulation and autoimmunity, so knocking it out lead to a forced activation of the immunity system, deleting the brakes that limit the immune response [54]. Similar trials with PD1‑knockout T cells for prostate, bladder cancer, as well as renal cell carcinoma, are also being initiated [55]. Other anticancer immune therapies are recently emerged founded on the production of next-generation chimeric antigen receptor (CAR) T cells [56]. These cells, which express tumor-targeting receptors, have shown promise in the treatment of various leukaemias, lymphomas and solid cancers. CARs include an extracellular binding domain which recognizes an antigen that is highly specific and strongly expressed on tumor cells, and an intracellular chimeric signalling domain that activates the T cells upon receptor engagement. This mechanism promotes T cell-mediated killing of tumor cells (Table 1).

**Table 1:** Novel gene editing-based therapies

Disease	Target	Reference
Duchenne Muscular Dystrophy	Dystrophin	[38–40]
Cystic Fibrosis	Cystic Fibrosis Transmembrane Conductance Regulator	[41]
HPV	E6 & E7	[42;43]
HBV	HBsAg & HBx	[44]
EBV	EBNA1, EBNA3C & LMP-1	[45]
	BART miRNA	[46]
AIDS	Gag/Pol - Rev/Env	[47]
	LTR-1, LTR-3, GagD & PolB	[48]
β-thalassaemia	β-/γ-globin	[49]
Leber congenital amaurosis	CEP290	[50]
Haemophilia	F9	[51]
Non-small Cell Lung Cancer	PD-1	[53]
Prostate Carcinoma	PD-1	[55]
Bladder Carcinoma	PD-1	[55]

On the left column it is reported the disease, on the central one the target gene and on the right one the reference number

In addition to the use described above, CRISPR-Cas system has the potential to be used to regulate endogenous gene expression or to label specific chromosomal loci in living cells or organisms. Catalytically inactive or “dead” Cas9 (dCas9) can be recruited by gRNAs to specific target DNA sites [57]. Targeting of dCas9 to promoters was initially shown to repress gene expression in both Escherichia coli and human cells [58–60]. It has also been demonstrated that an EGFP-dCas9 fusion can be used to visualize DNA loci harboring repetitive sequences, such as telomeres, with a single gRNA or non-repetitive loci using 26 to 36 gRNAs covering a 5-kb region of DNA [61]. This imaging system provides a powerful tool for studying chromosome dynamics and structure. It is also really interesting to see whether dCas9 fusions to histone modifiers and proteins involved in altering DNA methylation, can also be used to perform targeted “epigenome editing”. Indeed, it was recently found that many epigenetic factors are involved in multiple types of cancer such as glioblastoma, chondrosarcoma and osteosarcoma [62–64], so targeting epigenetic regulatory enzymes may be one suitable way to dysregulate tumor maintenance. As reported by Chen et al. [65], dCas9 could be fused to transcriptional activation domain such as VP64, VP64-p65-Rta, Kruppel-associated box (KRAB), activating or repressing selectively the target gene expression depending on the strength of activator or repressor used and the target transcription start site. In this way dCas9 might also be used to interfere with transcriptional elongation, directing to the non-template strand of a gene, resulting in 10- to 300- fold repression of mRNA transcription or when directed to a region which was initially occupied by RNA polymerase, dCas9 could also inhibit transcription initiation. CRISPR was also used to recreate mutations commonly identified in patients’ tumors and assess their effects in a cell line, to better understand the tumor-driving mutations, their phenotypic effects and then to identify new anticancer agents [66]. As reported by Matano et al. and Drost et al., it is possible to introduce a series of single mutations to transform an intestinal human organoid to an invasive carcinoma [67, 68] demonstrating that four mutations (APC, KRAS, SMAD4 and TP53) were mandatory to drive this process and that APC and TP53 loss was sufficient to induce chromosomal instability. Transducing a non-metastatic mouse lung cancer cell line with a lentiviral CRISPR/Cas9 library, targeting thousands of protein-coding genes and hundreds of microRNA precursors and inoculating these cells in immunodeficient mice, Chen et al. formed several growing tumors and lung metastases. The consequent deep sequencing analysis of the randomized KO tumor cells enabled the identification of several genes, whose inactivation may trigger tumor growth and invasion [69]. The main discovery of this kind of approach is that the selection of new tumor markers does not imply previous knowledge of the different functional hallmarks acquired throughout tumorigenesis. Another intriguing way to exploit CRISPR system would be to target tumor markers directly inside tumor site. In such a way it might be possible to overcome the genetic mutations leading tumor proliferation and metastatic capacity. However, in this approach the main problem is that every single tumor bears different mutation so it would require a very precise and personalized CRISPR therapy and another problem is the delivery system, because trying to make a selective gene knock out only in tumor cells is practically impossible with today technologies. An alternative target in cancer treatment for CRISPR might be the miRNAs expression. Indeed, miRNAs are involved in the regulation of a plethora of cellular physiological and pathological processes in a selective tissue-specific way [70], so the selective knock out of a single miRNA can lead to a powerful modulation of many genes at the same time. As demonstrated by Chang et al. the CRISPR-mediated knock out of miR-17, miR-200c and miR-141 in two colon cancer cell is actually possible and the repression could be stably maintained unaltered for a long term period [71]. So, it might be also possible to modulate miRNA expression with CRISPR technology in tumor-associated immune cells, regulating their recruitment and activation in the tumor microenvironment, waking up the immune system against tumor cells as yet demonstrated with the PD-1 KO [72]. The challenge for the future will be for sure to find a secure and safe way to delivery CRISPR machinery only in the tumor site and to inhibit its proliferative and metastatic ability. However, this system even if fascinating is not error free as yet reported above and so potential ethical concerns related to the impact of targeted nucleases upon cells germline are under discussion. Moreover, CRISPR application in vivo and especially in human has a very low working efficiency, demonstrating that we need to further improve our knowledge of this gene editing tool to create really efficient and safe therapy for all the disease related to a genetic mutation. Methods for expanding the targeting range of RNA-guided Cas9 will be important for inducing precise HDR or NHEJ events as well as for implementing multiplex strategies, including paired nickases.

## Conclusions

It is necessary to improve the reliability of the system in order to reduce off-target effects and improvements will be needed, particularly for therapeutic applications. Examples of such improvements might involve using protein engineering to modify Cas9 and/or modifying the nucleotides used by the gRNA to mediate recognition of the target DNA site. Alternatively, the construction of inducible forms of Cas9 and/or gRNAs might provide a means to regulate the active concentration of these reagents in the cell and thereby improve the ratio of on- and off-target effects. The simplicity, high efficiency and broad applicability of the RNA-guided Cas9 system have positioned this technology to transform biological and biomedical research. The ease with which researchers can now make changes in the sequence or expression of any gene means reverse genetics can be performed in virtually any organism or cell type of interest. All of these recent advances—and those to come—in developing and optimizing Cas9-based systems for genome and epigenome editing should propel the technology toward therapeutic applications, opening the door to treating a wide variety of human diseases.

## Abbreviations

AAV: Adeno-associated virus
Cas: CRISPR associated
CFTR: Cystic fibrosis transmembrane conductance regulator
CPP: Cell penetrating peptide
CRISPR: Clustered regularly interspaced short palindromic repeats
crRNA: CRISPR RNA
DSB: Double strand break
dsDNA: Double-stranded DNA
EBV: Epstein-barr virus
gRNA: Guide RNA
HBV: Hepatitis B virus
HDR: Homologous directed repair
HPVs: Human Papilloma viruses
KRAB: Kruppel-associated box
NHEJ: Non homologous end join
PAM: Protospacer adjacent motif
PD-1: Programmed cell death Protein – 1
ssDNA: Single-stranded DNA
ssODN: Single-stranded oligodeoxynucleotide
TALEN: Transcription activator-like effector nuclease
tracrRNA: Transactivating CRISPR RNA
ZFN: Zinc finger
